# A large choroid plexus cyst diagnosed with magnetic resonance imaging in utero: a case report

**DOI:** 10.4076/1757-1626-2-7098

**Published:** 2009-07-10

**Authors:** Mehdi Sasani, Ruya Afsharian, Hadi Sasani, Tunc Oktenoglu, Ali Fahir Ozer, Kemal Sarman

**Affiliations:** 1 Neurosurgery Department, American HospitalGuzelbahce Sk. No: 20, 34365 Nisantasi – IstanbulTurkey; 2 Obstetrics and Gynecology Department, Medamerican Medical CenterKadikoy-IstanbulTurkey; 3 Medicine Faculty, Radiology Department, Istanbul UniversityIstanbul-Turkey; 4 Istanbul Pathology CenterValikonagi Cd. No: 125, 34365 Nisantasi – IstanbulTurkey

## Abstract

The incidence of choroid plexus cysts represents approximately 1% of fetal anomalies. We describe a case in which fetal ultrasonography and fetal magnetic resonance scans were used to identify a large choroid cyst in a fetus without the use of a diagnostic amniocentesis to detect aneuploidy. After birth, the child underwent surgery. In conclusion, the nature of prenatal intracranial cysts should be fully evaluated and differentiated between choroid plexus cysts and other types of cysts. We believe that a detailed evaluation of detected cysts and other structural brain abnormalities are essential. Prenatal magnetic resonance scans clearly can decrease the need for risky procedures, such as an amniocentesis, in the evaluation of antenatal choroid plexus cysts.

## Introduction

The incidence of choroid plexus cysts (CPC) occurs in approximately 0.18% to 3.6% of routine fetal anomaly scans [1]. The diagnosing of CPCs using fetal ultrasonography (USG) was first defined in 1984 [2]. Although these cysts are normally benign, they may be associated with fetal aneuploidy [3].

Despite being described in numerous publications, management of a pregnancy in which a CPC has been found only by USG has engendered considerable controversy. The use of intrauterine magnetic resonance imaging (MRI) to evaluate CPCs is uncommon in comparison to USG. Here, we describe a case of a large CPC with fetal euploidy in an infant in which the lesion appeared on a fetal USG and MRI.

## Case presentation

**Figure 1.**
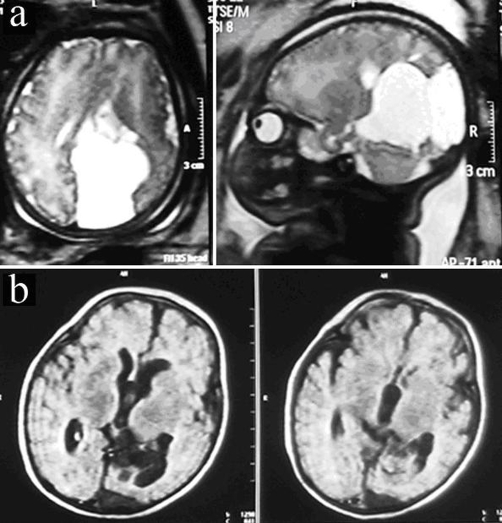
**(A)** A fetal MRI was showed multiseptal and subcortical cystic lesions on the right occipital lobe that originated from right lateral ventricle. **(B)** Annual MRI shown no recurrence of the CPC or septal membrane at post-operative 3 years.

A 26-year-old Turkish, white Caucasian healthy primigravida woman was referred for an antenatal anomaly found in an obstetrical ultrasound examination at 22 weeks of pregnancy. She was pregnant from at non-consanguineous marriage. The USG screening, showed a 10 mm in diameter cystic lesion on right occipital region with a slight hydrocephaly. A fetal MRI was performed to further evaluate the lesion at 26 weeks of pregnancy. Brain scans showed multiseptal and subcortical cystic lesions on the right occipital lobe that originated from right lateral ventricle (Figure 1A). Our first diagnosis was an occipital multiseptal arachnoid cyst. The patient was followed for another month with a USG once a week. Enlargement of the cyst was observed, but with no concomitant increase in cranial pressure. The patient experienced preterm labor pain at 32 weeks, resulting in the cesarean section delivery birth of a 1900 gm female.

**Figure 2.**
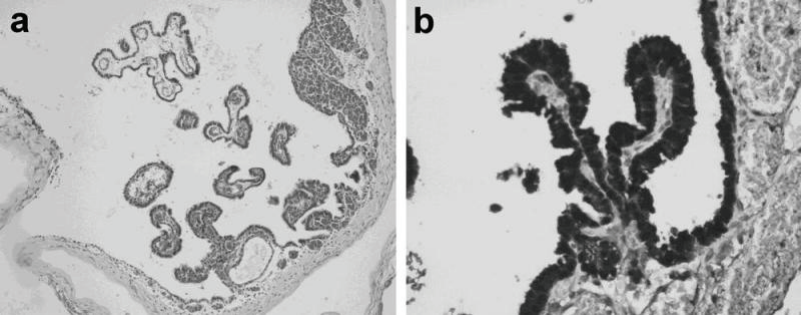
Histopathological examination revealed a CPC. **(A)** CPC is lined by one cell thick ependymal cells. Focally, it has converted into papillary fronds with highly vascular connective tissue cores. **(B)** The cells covering the fronds immonustain with S-100 protein but lack any evidence of immunoreactions with glial fibrillary acidic protein (GFAP).

The head circumference was 35 cm (within 2SD above the mean) and the weight was 3100 gm at 2 months of age. At 3 months, the head circumference increased to 40 cm (over 2SD above the mean) and the weight was 3850 gm. A cranial MRI showed an increase of intracranial pressure due to the dilatation of the lateral and 3rd ventricles. The infant underwent a small occipital craniotomy and resection of cyst membrane. Histopathological examination revealed a CPC (Figure 2).

That clinical examination revealed normal phenotype, chromosomal analysis confirmed normal genotype in neonate. Thus far we have followed the patient for 3 years, and annual MRI have shown no recurrence of the CPC or septal membrane (Figure 1B).

## Discussion

CPCs are cysts that occur within choroid plexus of the brain. The choroid plexus develops at approximately 6 weeks of gestation and begins producing cerebrospinal fluid, leading to the expansion of the ventricular system by the ninth week of gestation [4]. The accumulation of fluid can result in the formation of cysts, which can be detected by USG during the second trimester. CPCs have not been shown to impact neurodevelopment. They are typically temporary and usually resolved by 32^nd^ week gestation. For this reason large CPC as present case are very rare congenital malformations. Despite the low incidence, an important point that should be addressed is whether a chromosomal anomaly exists when there is a questionable lesion detected by USG. CPC have clinical implications for aneuploidy due to the association that the choroid plexus has with trisomy 18 and trisomy 21 [5]. In 44-50% of pregnancies with trisomy 18, antenatal USGs show CPCs, whereas only 1.4% of pregnancies show CPCs alone [6]. The physical characteristics of the USG, maternal age, and serum markers are three factors that contribute to the risk of aneuploidy. There is, however, consensus that while cysts with diameters less than 5 mm may not be linked to aneuploidy, large cysts in excess of 10 mm may carry a higher risk. In the presented case, we report a large CPC with observed normal chromosomal phenotype-genotype.

Many studies have concluded that the discovery of CPCs in otherwise normal fetuses does not by itself justify the risk of genetic amniocentesis, a subject with much controversy in the literature [7]. In the present case, the prenatal USG did not clearly detect the brain and the physical characteristics of the fetus, which can normally be visualized using MRI early in the second trimester.

In conclusion, the nature of prenatal intracranial cysts should be fully evaluated and differentiated between CPCs and other types of cysts. We believe that a detailed evaluation of detected cysts and other structural brain abnormalities are essential. Prenatal MR scans clearly can decrease the need for risky procedures, such as an amniocentesis, in the evaluation of antenatal CPCs.

Using prenatal USG associate with prenatal MR scans provide clearly assessment to show the physical characteristic (phenotype) of the fetus and fetus brain anatomy in CPC cases. This method is advisable to avoid of misdiagnosis in suspect cases who are been revealed with fetal USG.

## Abbreviations

CPC: Choroid Plexus Cysts
MRI: Magnetic Resonance Imaging
USG: Ultrasonography
